# Improving Scene Text Recognition for Indian Languages with Transfer Learning and Font Diversity

**DOI:** 10.3390/jimaging8040086

**Published:** 2022-03-23

**Authors:** Sanjana Gunna, Rohit Saluja, Cheerakkuzhi Veluthemana Jawahar

**Affiliations:** Centre for Vision Information Technology, International Institute of Information Technology, Hyderabad 500032, India; rohit.saluja@research.iiit.ac.in (R.S.); jawahar@iiit.ac.in (C.V.J.)

**Keywords:** scene text recognition, transfer learning, photo OCR, multi-lingual OCR, Indian languages, indic OCR, non-Unicode fonts, synthetic data

## Abstract

Reading Indian scene texts is complex due to the use of regional vocabulary, multiple fonts/scripts, and text size. This work investigates the significant differences in Indian and Latin Scene Text Recognition (STR) systems. Recent STR works rely on synthetic generators that involve diverse fonts to ensure robust reading solutions. We present utilizing additional non-Unicode fonts with generally employed Unicode fonts to cover font diversity in such synthesizers for Indian languages. We also perform experiments on transfer learning among six different Indian languages. Our transfer learning experiments on synthetic images with common backgrounds provide an exciting insight that Indian scripts can benefit from each other than from the extensive English datasets. Our evaluations for the real settings help us achieve significant improvements over previous methods on four Indian languages from standard datasets like IIIT-ILST, MLT-17, and the new dataset (we release) containing 440 scene images with 500 Gujarati and 2535 Tamil words. Further enriching the synthetic dataset with non-Unicode fonts and multiple augmentations helps us achieve a remarkable Word Recognition Rate gain of over 33% on the IIIT-ILST Hindi dataset. We also present the results of lexicon-based transcription approaches for all six languages.

## 1. Introduction

Multiple computer vision tasks rely on Scene Text Recognition (STR), and several commercial applications also benefit from it [1]. STR has diverse applications like helping the visually impaired, data mining of street-view-like images for information used in map services, and geographic information systems [2]. Conventionally, two steps are involved in STR. The first step is text detection, which consists of predicting word-level bounding boxes in the scene images [3]. The second step is text recognition, in which the Regions Of Interest (ROIs) obtained from the detection step are used to recognize the word text [4]. Our work involves improving text recognition in multiple Indian languages.

**Figure 1:**
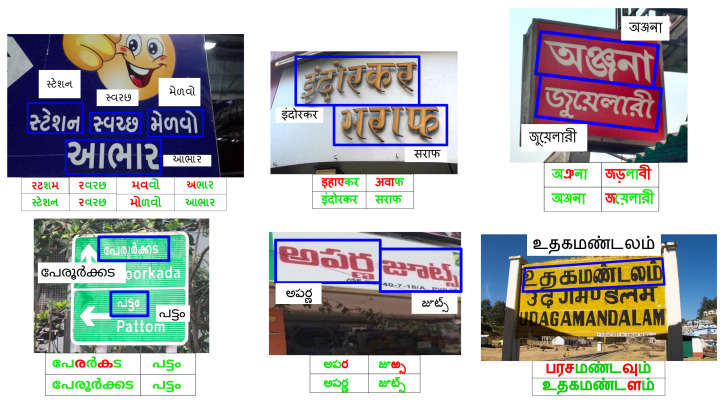
Clockwise from top-left: (**Top**(: Annotated Scene-text images, **Below each image**: Baselines’ predictions (row-1) and Transfer Learning models’ predictions (row-2), from Gujarati, Hindi, Bangla, Tamil, Telugu and Malayalam. Green, red, and “_” represent correct predictions, errors, and missing characters, respectively.

Human civilizations often involve reading multi-lingual text in scenes. The growth in non-Latin STR systems is gradual despite tremendous improvements in recognition models [5,6,7,8,9,10]. Developing a practical STR system for low resource languages remains challenging due to unstructured text appearing in diverse conditions such as scripts, fonts, sizes, and orientations. Moreover, recent scene-text recognition models are data-hungry and hence benefit from the use of synthetic data [4]. We also find synthetic data interesting to study the impact of transfer learning with the change in script or language text. We analyze such outcomes for transfer from English to Hindi and Gujarati. Our findings show that it is not beneficial to exploit the variety and scale in English datasets to improve the STR in Indian languages. We also perform experiments on the transferability of features among six different Indian languages and find that Indian languages can benefit each others’ STR systems. Our models further improve by using over 400 additional non-Unicode fonts in Devnagari to generate synthetic data. Rendering non-Unicode codes with synthetic engines requires additional efforts in creating translation tables which we will share with this work. Augmenting the synthetic dataset with methodologies proposed by et al. [11] further helps use create generalized STR systems for Indian languages. Figure 1 illustrates the sample annotated images from the six datasets from IIIT-ILST [4], MLT [12,13], and our work [14]. Below each image, we show the results from previous works followed by the result of our models. The contributions of this work are as:

1. We investigate the transfer learning of complete scene-text recognition models—(i) from English to two Indian languages (Hindi and Gujarati) and (ii) among the six Indian languages, i.e., Gujarati, Hindi, Bangla, Telugu, Tamil, and Malayalam.
2. We also contribute two datasets of around 500 word images in Gujarati and 2535 word images in Tamil from a total of 440 Indian scenes.
3. On IIIT-ILST Hindi, Telugu, and Malayalam datasets, we achieve gains of 37%, 5%, and 2% in Word Recognition Rates (WRRs) over prior works [4,15]. We observe the WRR gains of 8%, 4%, 5%, and 3% over our baseline model on the MLT-19 Hindi and Bangla datasets as well as Gujarati and Tamil datasets we release, respectively. Our model also achieves a notable WRR gain of 24% for the MLT-17 Bangla dataset compared to Bušta et al. [2].
4. We are the first to use over 500 Hindi fonts with an existing synthesizer and show that the font diversity can significantly improve non-Latin STR. Specifically, on the IIIT-ILST Hindi dataset, we achieve a remarkable WRR gain of over 33% using more than 500 fonts and different augmentation techniques.

## 2. Related Work

We now discuss datasets and associated works in the field of photo-OCR.

**Works of Photo-OCR on Latin Datasets:** As stated earlier, the process of Photo-OCR conventionally includes two steps: (i) Text detection and (ii) text recognition. With the success of Convolutional Neural Networks (CNN) for object detection, the works have been extended to text detection, treating words or lines as the objects [16,17,18]. Liao et al. [19] extend such works to real-time detection in scene images. Karatzas et al. [20] and Bušta et al. [21] present more efficient and accurate methods for text detection. Towards reading scene-text, Wang et al. [22] propose an object recognition pipeline based on a ground truth lexicon. It achieves competitive performance without the need for an explicit text detection step. Shi et al. [23] propose a Convolutional Recurrent Neural Network (CRNN) architecture, which integrates feature extraction, sequence modeling, and transcription into a unified framework. The model achieves remarkable performances in both lexicon-free and lexicon-based scene-text recognition tasks. Liu et al. [24] introduce Spatial Attention Residue Network (STAR-Net) with spatial transformer-based attention mechanism to remove image distortions, residue convolutional blocks for feature extraction, and an RNN block for decoding the text. Shi et al. [10] propose a segmentation-free Attention-based method for Text Recognition (ASTER) by adopting Thin-Plate-Spline (TPS) as a rectification unit. It tackles complex distortions and reduces the difficulty of irregular text recognition. The model incorporates ResNet to improve the network’s feature representation module and employs an attention-based mechanism combined with a Recurrent Neural Network (RNN) to form the prediction module. Uber-Text is a large-scale Latin dataset that contains around 117 K images captured from 6 US cities [25]. The images are available with line-level annotations. The French Street Name Signs (FSNS) data contains around 1000 K annotated images, each with four street sign views. Such datasets, however, contain text-centric images. Reddy et al. [26] recently release RoadText-1K to introduce challenges with generic driving scenarios where the images are not text-centric. RoadText-1K includes 1000 video clips (each 10 s long at 30 fps) from the BDD dataset, annotated with English transcriptions [27]. Ghosh et al. [28] proposes a dataset consisting 1154 images of movie posters spanning over multiple scripts.

**Works of Photo-OCR on Non-Latin Datasets:** Recently, there has been an increasing interest in scene-text recognition for non-Latin languages such as Chinese, Korean, Hindi, Japanese, etc. Several datasets like RCTW (12 K scene images), ReCTS-25k (25 K signboard images), CTW (32 K scene images), and RRC-LSVT (450 K scene images) from ICDAR’19 Robust Reading Competition (RRC) exist for Chinese [29,30,31,32]. Arabic datasets like ARASTEC (260 images of signboards, hoardings, and advertisements) and ALIF (7 K text images from TV Broadcast) also exist in the scene-text recognition community [33,34]. Korean and Japanese scene-text recognition datasets include KAIST (2385 images from signboards, book covers, and English and Korean characters) and DOST (32 K sequential images) [35,36]. The MLT dataset available from the ICDAR’17 RRC contains 18 K scene images (around 1–2 K images per language) in Arabic, Bangla, Chinese, English, French, German, Italian, Japanese, and Korean [12]. The ICDAR’19 RRC builds MLT-19 over top of MLT-17 to contain 20 K scene images containing text from Arabic, Bangla, Chinese, English, French, German, Italian, Japanese, Korean, and Hindi [13]. The RRC also provides 277 K synthetic images in these languages to assist the training. Singh et al. [37] propose a large and diverse OCR dataset with over 1 M annotations including multilingual scene text data. Mathew et al. [4] train the conventional encoder-decoder, where Convolutional Neural Network (CNN) encodes the word image features. An RNN decodes them to produce text on synthetic data for Indian languages. Here an additional connectionist temporal classification (CTC) layer aligns the RNN’s output to labels. The work also releases an IIIT-ILST dataset for testing that reports Word Recognition Rates (WRRs) of 42.9%, 57.2%, and 73.4% on 1 K real images in Hindi, Telugu, and Malayalam, respectively. Bušta et al. [2] proposes a CNN (and CTC) based method for text localization, script identification, and text recognition. The model is trained and tested on 11 languages of MLT-17 dataset. The WRRs are above 65% for Latin and Hangul and are below 47% for the remaining languages. The WRR reported for Bengali is 34.20%. Recently, an OCR-on-the-go model and obtain the WRR of 51.01% on the IIIT-ILST Hindi dataset and the Character Recognition Rate (CRR) of 35% on a multilingual dataset containing 1000 videos in English, Hindi, and Marathi [15]. Around 2322 videos in these languages recorded with controlled camera movements like tilt, pan, etc., are additionally shared. Ghosh et al. propose a LWSINet [38] for video data and a shallow convolutional neural network (SCNN)-based architecture [39] for image data for script identification allows improved functionality over low-resource scripts, especially Indic scripts. Huang et al. [40] propose a scalable end-to-end trainable Multilingual Mask TextSpotter, which optimizes script identification while maintaing multiple recognition heads for different scripts.

**Transfer Learning in Photo-OCR:** With the advent of deep learning in the last decade, transfer learning became an essential part of vision models for tasks such as detection and segmentation [41,42]. The CNN layers pre-trained from the Imagenet classification dataset are conventionally used in such models for better initialization and performance [43]. The scene-text recognition works also use the CNN layers from the models pre-trained on Imagenet dataset [10,23,24]. Ghosh et al. [28] proposes a transfer learning-based approach for graphic-rich text localization whereas we focus on text recognition. However, to our best knowledge, there are no significant efforts on transfer learning from one language to another in the field of scene-text recognition, although transfer learning seems to be naturally suitable for reading low resource languages. We investigate the possibilities of transfer learning in all the layers of deep photo-OCR models.

## 3. Motivation

We now discuss the motivation for our work.

**Figure 2:**
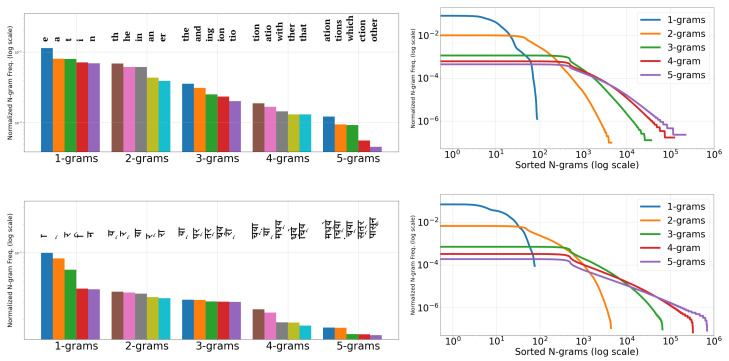
Distribution of Char. n-grams (n∈[1,5]) from 2.5 M words in English and Hindi (**top** to **bottom**): Top-5 (**left**) and All (**right**).

**Transfer Learning amongst Indian scripts:** As discussed earlier in Section 2, most of the scene-text recognition works use the pre-trained Convolutional Neural Networks (CNN) layers for improving results. We now motivate the need for transfer learning of the complete recognition models discussed in Section 1 and the models we use in Section 5 among different languages. As discussed in these sections, the Recurrent Neural Networks (RNNs) form another integral component of such reading models. Therefore, we illustrate the distribution of character-level n-grams they learn in Figure 2 (For plots on the right, we use moving averages of 10, 100, 1000, 1000, 1000 for 1-grams, 2-grams, 3-grams, 4-grams, and 5-grams, respectively) for the first five languages we discussed in the previous section (we notice that the last two languages also follow a similar trend). On the left, we show the frequency distribution of top-5 n-grams, (n∈[1,5]). On the right, we show the frequency distribution of all n-grams with n∈[1,5]. We use 2.5 M words from English and Hindi for these plots. The total number of English letters is of the same order as Indian languages. The x-values (≤100) for the drops in 1-gram plots (blue curves) of Figure 2 also illustrates this. So, it becomes possible to compare the distributions. The overall distributions are similar for all the languages. Hence, we propose that the RNN layers’ transfer among the models of different languages is worth an investigation.

It is essential to note the differences between the n-grams of English and Indian languages. Many of the top-5 n-grams in English are the complete word forms, which is not the case with Indian languages owing to their richness in inflections (or fusions) [44]. Additionally, note that the second 1-gram for Hindi in Figure 2 (left), known as halanta, is a common feature of top-5 n-grams in Indian languages. The halanta forms an essential part of joint glyphs or aksharas (as advocated by Vinitha et al. [44]). In Figure 1, the vowels, or portions of the joint glyphs for word images in Indian languages, often appear above the top-connector line or below the generic consonants. All this, in addition to complex glyphs in Indian languages, makes transfer learning from English to Indian languages ineffective, which is detailed in Section 7. Thus, we also investigate the transferability of features among the Indian scene-text recognition models in the subsequent sections.

**Figure 3:**
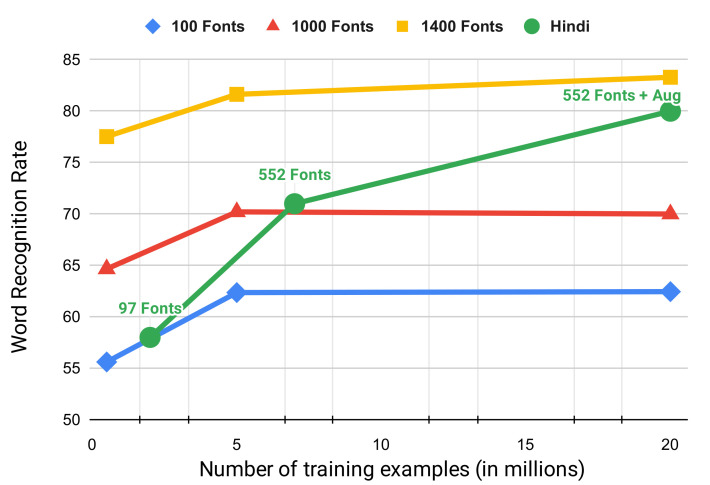
Comparing STAR-Net’s performance on IIIT5K [48] dataset (blue, red, and yellow plots) and IIIT-ILST [4] Hindi dataset (green plot) when trained on synthetic data created using a varying number of fonts and training samples.

**Significance of the variety of fonts:** To study the effect of fonts and training examples, we extend the analysis in Gunna et al. [45] on English fonts obtained from previous works [46,47] for Hindi fonts we use in this work. The results are shown in Figure 3. We observe that on the IIIT5K dataset, with 100 and 1000 fonts (blue and red plots), the WRR increase when the number of samples increases from 0.5 M to 5 M and then becomes constant for 20 M samples. However, the increase in WRR on the IIIT5K dataset is significant when the number of fonts increases from 1000 to 1400 (blue plot). The higher font diversity (1400 fonts) also helps in improving the results with an increase in the number of samples from 5 M to 20 M.

For the IIIT-ILST Hindi dataset, we use three settings for our experiment: 2 M samples with 97 fonts, 7 M samples with 552 fonts, and augmented 20 M samples with 552 Fonts for our analysis. The results are shown in the green plot in Figure 3. Increasing the fonts from 97 to 552 and training samples from 2 M to 7 M improve the Hindi WRR from 58% to 71% (similar to English). We also reduce the WRR gap due to 552 Hindi fonts and 1400 Latin fonts by applying augmentations discussed in Section 5. As we will see in Section 5, the stretching/compressing augmentation deals with varying the width of image parts, thus creating an effect like having multiple fonts in a word image. We, therefore, observe that WRR gains due to augmentation in Hindi are similar to increased fonts and training samples in Latin. Finally, we achieve the WRR of over 80% for the IIIT-ILST Hindi dataset with augmented 20 M samples created using 552 Fonts.

## 4. Datasets

**Synthetic Datasets:** As shown in Table 1, we generate 2.5 M, or more, word images each in Hindi, Bangla, Tamil, Telugu, and Malayalam (For Hindi, Telugu and Malayalam, our models trained on 2.5 M word images achieved results lower than previous works, so we generate more examples equal to (and to fairly compare with) Mathew et al. [4].) with the methodology proposed by Mathew et al. [4]. For each Indian language, we use 2 M images or more for training our models and the remaining set for testing.

**Figure 4:**
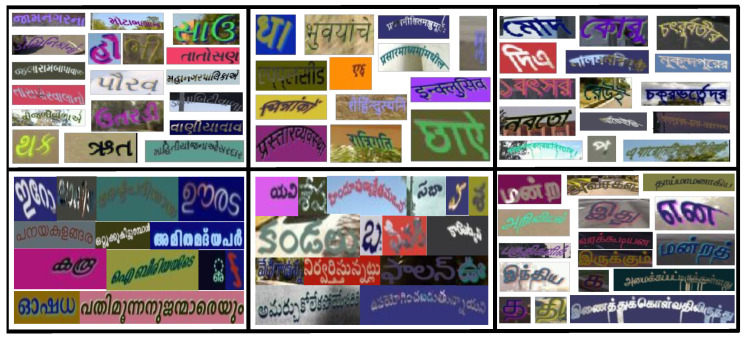
Clockwise from top-left: synthetic word images in Gujarati, Hindi, Bangla, Tamil, Telugu, and Malayalam. Notice that a top-connector line connects the characters to form a word in Hindi or Bangla. Some vowels and characters appear above and below the generic characters in Indian languages, unlike English.

Sample images of our synthetic data are shown in Figure 4. For English, we use the models pre-trained on the 9 M MJSynth and 8 M SynthText images [46,47]. We generate 0.5 M synthetic images in English with over 1200 fonts for testing. As shown in Table 1, English has a lower average word length than Indian languages. We list the Indian languages in the increasing order of language complexity, with visually similar scripts placed consecutively, in Table 1. Gujarati is chosen as the entry point from English to Indian languages as it has the lowest word length among all Indian languages. Subsequently, like English, Gujarati does not have a top-connector line that connects different characters to form a word in Hindi and Bangla (refer to Figure 1 and Figure 4).

Additionally, the number of Unicode fonts available in Gujarati is fewer than those available in other Indian languages. Next, we choose Hindi, as Hindi characters are similar to Gujarati characters and the average word length of Hindi is higher than Gujarati. Bangla has comparable word length statistics with Hindi and shares the property of the top-connector line with Hindi. Still, we keep it after Hindi in the list as its characters are visually dissimilar and more complicated than Gujarati and Hindi. We use less than 100 for fonts in Hindi, Bangla, and Telugu. We list Tamil after Bangla because these languages share similar vowels’ appearance (see the glyphs above general characters in Figure 4). Tamil and Malayalam have the highest variability in word length and visual complexity compared to other languages. Please note that we have over 150 fonts available in Tamil.

**Figure 5:**
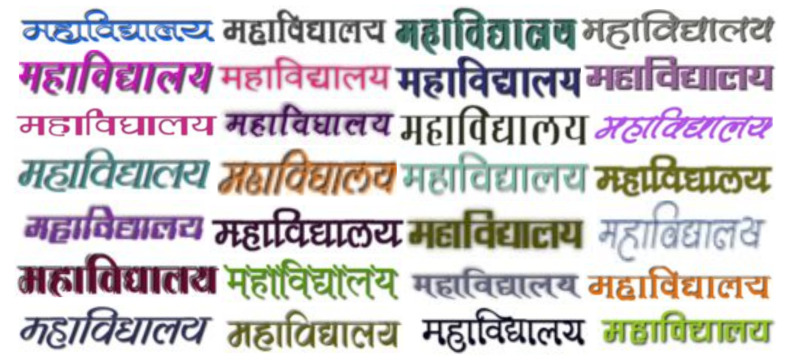
Examples of non-Unicode Hindi fonts used for synthetic data generation.

To test the role of fonts in scene-text recognition performance, we collect 455 additional non-Unicode fonts, through a region-based search, for experiments in Hindi. Using non-Unicode fonts in the data generation process is difficult because of their non-existent Unicode equivalents. Even most of the recent synthetic data generation engines [47,49,50] do not have a fix to employ non-Unicode fonts properly, especially for non-Latin languages. We create table-based converters to convert the non-Unicode characters to Unicode text. However, much work went into effectively handling the matras (e.g., vowel signs and halanta) in the language, as they change based on their positions. These mappings help utilize all the fonts to render synthetic images without errors (like incorrectly ordered joint glyphs or empty box images instead of characters). Sample images of a few of the fonts that went into the training process are shown in Figure 5. We combine the Unicode fonts (as mentioned in Table 1) and the new non-Unicode fonts to create synthetic data for the experiments based on the number of fonts. We generate 7 M samples with a total of 552 fonts (Unicode and non-Unicode) with the same vocabulary mentioned previously.

**Real Datasets:** We also perform experiments on the real datasets from IIIT-ILST, MLT-17, and MLT-19 datasets (refer to Section 2 for these datasets). To enlarge scene-text recognition research in complex and straight forward low-resource Indian Languages, we release 500 and 2535 annotated word images in Gujarati and Tamil. We crop the word images from 440 annotated scene images, which we obtain by capturing and compiling Google images. We illustrate sample annotated images of different datasets in Figure 1. Similar to MLT datasets, we annotate the Gujarati and Tamil datasets using four corner points around each word (see Tamil image at bottom-right of Figure 1). IIIT-ILST dataset has two-point annotations leading to an issue of text from other words in the background of a cropped word image as shown in the Telugu scene at the bottom-middle of Figure 1.

## 5. Methodology

This section explains the two models we use for transfer learning in Indian languages, a plug-in module we propose for learning the correction mechanism in the recognition systems and an augmentation pipeline to improve data diversity.

**Figure 6:**
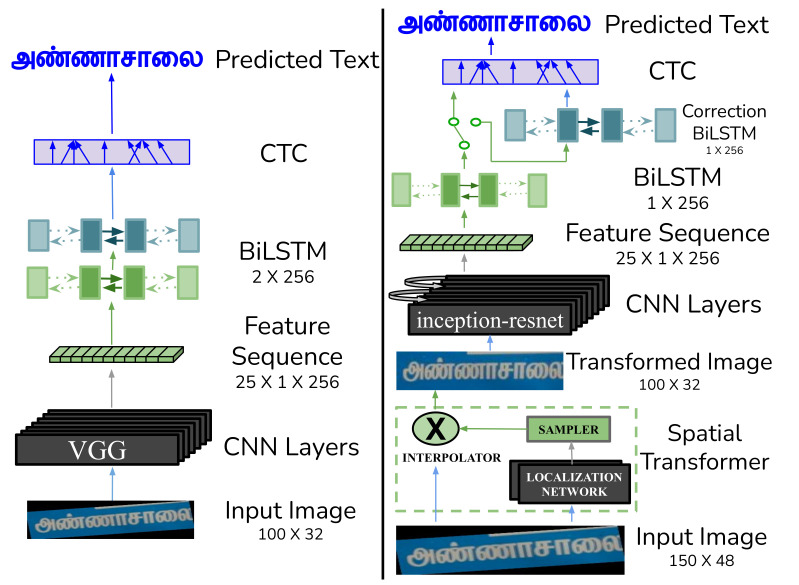
CRNN model (**left**) and STAR-Net with a correction BiLSTM (**right**).

**CRNN Model (Baseline 1):** The first baseline model we train is Convolutional-Recurrent Neural Network (CRNN), which is the combination of CNN and RNN as shown in Figure 6 (left). The CRNN network architecture consists of three fundamental components, (i) an encoder composed of the standard VGG model [51], (ii) a decoder consisting of RNN, and (iii) a Connectionist Temporal Classification (CTC) layer to align the decoded sequence with ground truth. The CNN-based encoder consists of seven layers to extract feature representations from the input image. The model abandons fully connected layers for compactness and efficiency. It replaces standard squared pooling with 1×2 sized rectangular pooling windows for 3rd and 4th max-pooling layer to yield feature maps with a larger width. A two-layer Bi-directional Long Short-Term Memory (BiLSTM) model, each with a hidden size of 256 units, then decodes the features. The CTC layer provides non-parameterized supervision during the training phase to align the decoded predictions with the ground truth. We use greedy decoding during the testing time. We use the PyTorch implementation of the model by Shi et al. [23].

**STAR-Net (Baseline 2):** As shown in Figure 6 (right), the STAR-Net model (our second baseline model) consists of three components, (i) a Spatial Transformer to handle image distortions, (ii) a Residue Feature Extractor consisting of a residue CNN and an RNN, and (iii) a CTC layer to align the predicted and ground truth sequences. The transformer consists of a spatial attention mechanism achieved via a CNN-based localization network, a sample, and an interpolator. The localizer predicts the parameters of an affine transformation. The sampler and the nearest-neighbor interpolator use the transformation to obtain a better version of the input image. The transformed image acts as the input to the Residue Feature Extractor, which includes the CNN and a single-layer BiLSTM of 256 units. The CNN used here is based on the inception-resnet architecture, which can extract robust image features required for the task of scene-text recognition [52]. The CTC layer finally provides the non-parameterized supervision for text alignment. The overall model consists of 26 convolutional layers and is end-to-end trainable [24].

**Transfer Learning:** We initially train individual models for each language to analyze the performance and the features learned by the models for further transfers. For the transfer learning experiments among languages, we finetune all network layers.

**Correction BiLSTM:** After training the STAR-Net model on a real dataset, we add a correction BiLSTM layer (of size 1×256), an end-to-end trainable module, to the end of the model (see Figure 6 top-right). We re-train the complete model on the same dataset to implicitly learn the error correction mechanism.

**Augmentation Pipeline:** As discussed in the previous section, we use 455 additional fonts to increase font diversity in the synthetic training dataset for Hindi. We apply augmentations to the synthetic data for inducing more variety into the training samples. It also helps prevent the model from over-fitting. We use a total of 9 augmentation techniques as proposed by [11], which include blur, noise, distortion, inversion, curving, rotation, stretching/compressing, perspective, and shrinking. As discussed earlier in Section 3, the stretching/compressing augmentation creates an effect similar to using multiple fonts in a single image and hence improves the font diversity further. We sample three augmentation functions from nine and apply them one after the other to each image in the batch. We avoid using all nine augmentations on a single image as it will cause indistinguishable damage to the input. For each image, we produce three augmented images. In addition to augmented images, we also use original word images in our training samples. A few of the techniques mentioned above change the image dimensions, so we resize the image to the input sizes of the baseline models while maintaining the aspect ratio.

## 6. Experiments

The images, resized to 150×48, form the input of STAR-Net, which we refer to as baseline 2. The spatial transformer module, as shown in Figure 6 (right), then outputs the image of size 100×32. The size of inputs to the CNN Layers of baseline 1 (CRNN) and baseline 2 are the same, i.e., 100×32, and the common output size is 25×1×256. The localization network in baseline 2 has four plain convolutional layers with 16, 32, 64, and 128 channels. Each layer has the filter size, stride, and padding size of 3, 1, and 1, followed by a 2×2 max-pooling layer with a stride of 2. Finally, a fully connected layer of size 256 outputs the parameters which transform the input image. We train all our models on 2 M or more synthetic word images as discussed in Section 4. We use the batch size of 16 and the ADADELTA optimizer for stochastic gradient descent (SGD) for all the experiments [53]. Epochs vary between 10 to 15 for different experiments. We test our models on 0.5 M synthetic images for each language. We use the word images from IIIT-ILST, MLT-17, and MLT-19 datasets to analyze the performance on real datasets.

We fine-tune the Bangla models on 1200 training images and test them on 673 validation images from the MLT-17 dataset to fairly compare with Bušta et al. [21]. Similarly, we fine-tune only our best Hindi transfer learning model on the MLT-19 dataset and test it on the IIIT-ILST dataset to compare with OCR-on-the-go (since it is also trained on real data) [15]. To demonstrate generalizability, we also test our models on 3766 Hindi images and 3691 Bangla images available from MLT-19 datasets [13]. For Gujarati and Tamil, we use 75% of word images to fine-tune our models and the remaining 25% for testing. Our experiments include transfer learning from English to two Indian languages (Gujarati and Hindi) and transfer learning among the six Indian languages. As discussed in the previous section, we also perform experiments with Correction BiLSTM in Bangla and multiple fonts and augmentations in Hindi. We finally improve present the improvements in results for all six Indian languages with lexicon-based transcription approaches [22,48,54].

## 7. Results

In this section, we discuss the results of our experiments with (i) individual models for each language, (ii) the transfer learning from English to two Indian languages, (iii) the transfer learning among six Indian languages, (iv) correction BiLSTM, (v) increasing font diversity with augmentations, and (vi) lexicon-based transcription approaches.

**Performance on Synthetic Datasets:** It is essential to compare the results on synthetic datasets of different languages sharing common backgrounds, as it provides a good intuition about the difficulty in reading different scripts. In Table 2 and Table 3, we present the results of our individual languages and transfer learning experiments with synthetic datasets respectively. As noted in Table 2, the baseline 1 model achieves the Character Recognition Rates (CRRs) and Word Recognition Rates (WRRs) of (i) 77.13% and 38.21% in English and (ii) above 82% and 48% on the synthetic dataset of all the Indian languages (refer to columns 1 and 2 of Table 2). The low accuracy on the English synthetic test set is due to the presence of more than 1200 different fonts (refer Section 4). Nevertheless, using a large number of fonts in training helps in generalizing the model for real settings [46,47]. The baseline 2 achieves remarkably better performance than baseline 1 on all the datasets, with the CRRs and WRRs above 90.48 and 65.02 for Indian languages. The reason for this is spatial attention mechanism and powerful residual layers, as discussed in Section 5. As shown in columns 3 and 5 of Table 2, the WRR of the models trained in Gujarati, Hindi, and Bangla are higher than the other three Indian languages despite common backgrounds. The experiments show that the scripts in latter languages pose a tougher reading challenge than the scripts in former languages.

We present the results of our transfer learning experiments on the synthetic datasets in Table 3. The best individual model results from Table 2 are included in parenthesis for comparison. We begin with the English models as the base because the models have trained on over 1200 fonts and 17 M word images as discussed in Section 4, and are generic. However, in the first two rows of the table, we note that transferring the layers from the model trained on the English dataset to Gujarati and Hindi is inefficient in improving the results compared to the individual models. The possible reason for the inefficiency is that Indic scripts have many different visual and slightly different n-gram characteristics from English, as discussed in Section 4. We then note that as we try to apply transfer learning among Indian languages with baseline 1 (rows 3–7, columns 1–2 in Table 3), only some combinations work well. However, with baseline 2 (rows 3–7, columns 3–4 in Table 3), transfer learning helps improve results on the synthetic dataset from a simple language to a complex language (We also discovered experiments on transfer learning from a tricky language to a simple one to be effective, but slightly less than the reported results.). For Malayalam, we observe that the individual baseline 2 model is better than the one transferred from Telugu, perhaps due to high average word length (refer Section 4).

**Performance on Real Datasets: **Table 4 depicts the performance of our models on the real datasets. At first, we observe that for each Indian language, the overall performance of the individual baseline 2 model is better than the individual baseline 1 model (except for Gujarati and Hindi, where the results are very close). Based on this and similar observations in the previous section, we present the results of transfer learning experiments on real datasets with only the baseline 2 model (We also tried transfer learning with baseline 1; baseline 2 was more effective.). Next, similar to the previous section, we observe that the transfer learning from English to Gujarati and Hindi IIIT-ILST datasets (rows 3 and 8 in Table 4) is not as effective as individual models in these Indian languages (rows 2 and 7 in Table 4). Finally, we observe that the performance improves with the transfer learning from a simple language to a complex language, except for Hindi→Gujarati, for which Hindi is the only most straightforward choice. We achieve performance better than the previous works, i.e., Bušta et al. [21], Mathew et al. [4], and OCR-on-the-go [15]. Overall, we observe the increase in WRRs by 6%, 5%, 2% and 23% on IIIT-ILST Hindi, Telugu, and Malayalam, and MLT-17 Bangla datasets compared to the previous works. On the MLT-19 Hindi and Bangla datasets, we achieve gains of 8% and 4% in WRR over the individual baseline 1 models. On the datasets we release for Gujarati and Tamil, we improve the baselines by 5% and 3% increase in WRRs. We present the qualitative results of our baseline 1 models as well as best transfer learning models in Figure 1. The green and red colors represent the correct predictions and errors, respectively. “_” represents the missing character. As can be seen, most of the mistakes are single-character errors.

**Performance with Correction BiLSTM:** Since we observe the highest gain of 23% in WRR (and 4% in CRR) for the MLT-17 Bangla dataset (Table 4), we further try to improve these results. We plug in the correction BiLSTM (refer Section 5) to the best model (row 18 of Table 4). The results are shown in row 19 of Table 4. As shown, the correction BiLSTM improves the CRR further by a notable margin of 11% since the BiLSTM works on character level. We also observe the 1% WRR gain, thereby achieving the overall 24% WRR gain (and 15% CRR gain) over Bušta et al. [21].

**Figure 7:**
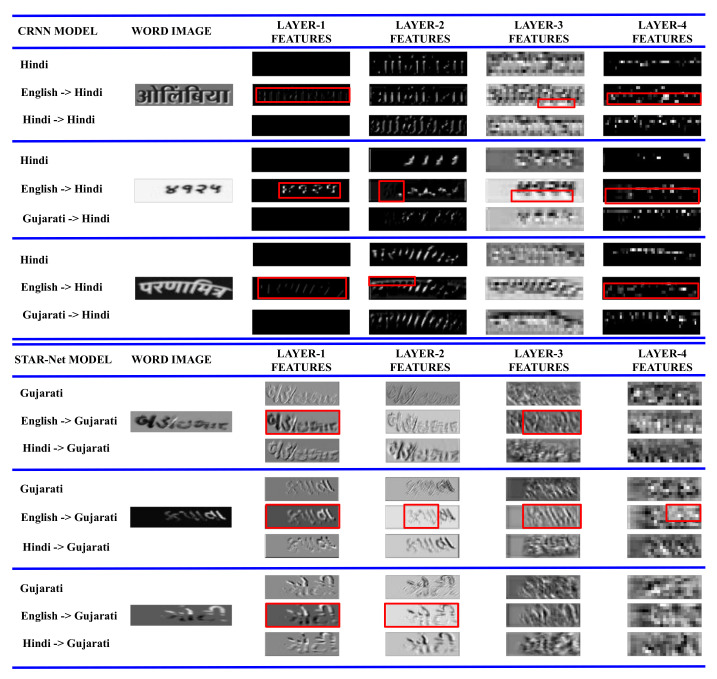
CNN layers visualization in the (**Top**): Baseline 1 (CRNN) models trained on Hindi, English→Hindi, and Gujarati→Hindi; and (**Bottom**): Baseline 2 (STAR-Net) models trained on Gujarati, English→Gujarati, and Hindi→Gujarati. Red boxes indicate the regions where the features for the model transferred from English are activated (as white), whereas the features from the other two models are not.

**Features Visualization:** In Figure 7 for the baseline 1 model (top three triplets), we visualize the learned CNN layers of the individual Hindi model, the “English→Hindi” model, and the “Gujarati→Hindi” model. The red boxes are the regions where the first four CNN layers of the model transferred from English to Hindi are different from the other two models. The feature visualization again strengthens our claim that transfer from the English reading model to any Indian language dataset is inefficient. We notice a similar trend for the Gujarati baseline 2 models, though the initial CNN layers look very similar to word images (bottom three triplets in Figure 7). The similarity also demonstrates the better learnability of baseline 2 compared to baseline 1, as observed in previous sections. 

**Incorporating more fonts and augmentation techniques:** We present results of the baseline 2 experiments with 552 fonts in Table 5. For the IIIT-ILST Hindi dataset, the model trained on 7 M samples generated with 552 fonts (row 1, Table 5) shows an improvement of 9.28% and 24.85% in CRR and WRR, respectively, over the model trained on 97 fonts (row 2, Table 5). Similarly, for the MLT-19 Hindi dataset, we observe 1.35% and 1.33% CRR and WRR gains, respectively, despite high CRR (86.53%) of the model trained on 97 fonts (row 4, Table 5). Hence, it supports our claim that the number of fonts used to create the synthetic dataset plays a crucial role in improving the scene text recognition models.

**Figure 8:**
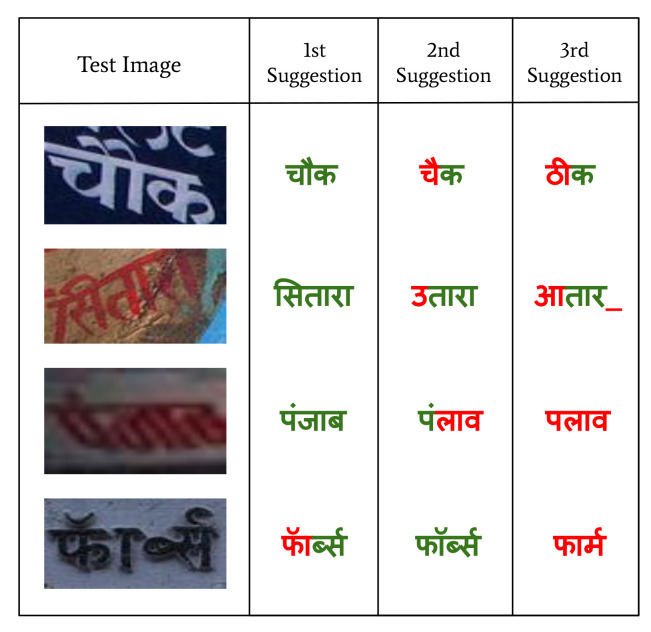
Top-3 suggestions of our improved Hindi model with fonts and augmentation on Hindi IIIT-ILST dataset. The green and red colors represent the correct predictions and errors, respectively. “_” represents the missing character.

**Figure 9:**
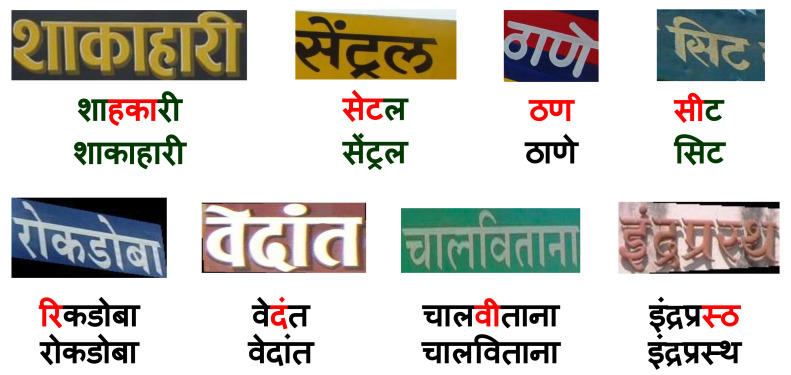
Examples of real images from IIIT-ILST (**top**) and MLT-19 (**bottom**) Hindi datasets. Below the images—(i) Baseline 2 predictions, (ii) correct predictions by Baseline 2 model with increased fonts and augmentation. Text in red represents errors.

Further, using 2 M synthetic images, we generate 8 M training samples (2 M original and 6 M augmented) by incorporating the augmentation pipeline described in Section 5. Note that the order of the number of augmented samples (8 M) is similar to non-augmented data (7 M samples) in rows 1, 2, 4, and 5 in Table 5 (We also apply the augmentation pipeline over the 7 M synthetic data, generating over 20 M training samples, and do not observe accuracy improvements. Refer Section 3 and Figure 3 for results with 20 M augmented training samples in Hindi.). We can note that applying the augmentation techniques to the synthetic data helps a lot compared to the baseline 2 models trained on non-augmented 7 M samples (refer to the rows 2,3 and rows 5,6 of Table 5). Though the number of training samples is similar in both cases, the model with augmentation pipeline produced better results than the baseline 2 model. The augmentation has shown striking CRR and WRR gains of 2.92% and 8.88% over the model without any augmented data (refer to row 2 of Table 5) for the IIIT-ILST dataset. Moreover, augmentation also shows an improvement of 5.65% in WRR for MLT-19 Hindi (compare rows 5 and 6 of Table 5). It is also important to note that we achieve the CRRs of above 90% for the two Hindi datasets (IIIT-ILST and MLT-19). We showcase the interactivity of our proposed Hindi model in terms of suggestions in Figure 8. We present the top-3 suggestions offered by our models to the test samples in the case of lexicon-based transcription. The first suggestion is usually the correct prediction but in case if the first suggestion is incorrect, second or third suggestions might be correct (refer the last image in Figure 8 where the spelling in the image is incorrect and the second suggestion is the correct prediction instead of the first). We present the qualitative results of our fonts and augmentation experiments on IIIT-ILST Hindi and MLT-19 Hindi in Figure 9. Below each image, we show the prediction from baseline 2, followed by the upgraded model trained with data synthesized using additional fonts and augmentations. It is interesting to note in the three samples on the top-right and three samples on the bottom-left of Figure 9, that the upgraded model can handle conjunct characters (with matras or vowels) more efficiently than the baseline model.

**Performance with lexicon-based transcription:** Lexicon-based transcriptions achieve better results on the datasets [22,48,54]. We created two lexicons for the datasets to achieve better performance. Each test sample has a small lexicon containing 50 words. It includes the ground-truth word and other distractor words from the test set. The large lexicon contains 1000 words, and it includes most of the label set and the most frequently used words in the language to sum it all up to 1000. We used the BK-tree data structure [55], a metric tree specifically adapted to discrete metric spaces to store the lexicon for fast searching. We test our models by finding words in the tree data structure with ≤3 edit distance to the query label. The usage of small and large lexicons during post-processing performs better than the lexicon-free based testing. We test our best models using the lexicon-based approaches and present the results in Table 6. For Hindi, we present the results of our best model, baseline 2, with augmentation using the lexicon-based transcription. We observe an increase of around 8.5% and 18.5% in CRR and WRR (refer to row 2 of Table 6), respectively, with a large lexicon compared to the lexicon-free approach on the IIIT-ILST Hindi dataset. Similarly, as shown in row 3 of Table 6, we observe a 25.92% increase in WRR on the MLT-19 Hindi dataset for the small (50) lexicon-based transcription. We observe similar improvements in the performance for other languages as well. The results on the Gujarati dataset reach a maximum of 100% for both WRR and CRR for the small lexicon-based transcription because of the high CRR of the lexicon-free model (90.82) and ineffective 50 distractors randomly chosen from 100 unique labels in the test set.

**Figure 10:**
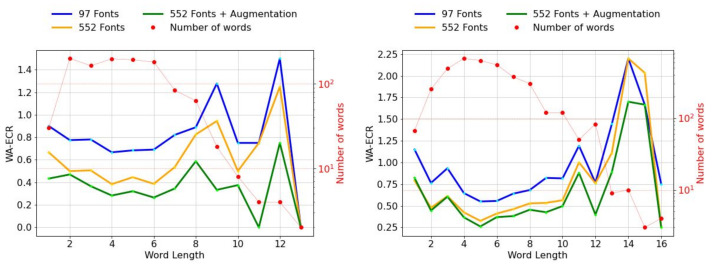
WA-ECR of our Hindi models tested on IIIT-ILST (**left**) and MLT-19 (**right**) datasets.

**Error Analysis:** We analyze the quantitative results using two tools of evaluation, i.e, WA-ECR plots (Word-Averaged Erroneous Character Rate) and histogram plots [56]. We present the WA-ECR plots on Hindi real datasets in Figure 10. WA-ECR values are usually lower for longer length words because of their low numbers. However, lower values of WA-ECR also indicate the decrease in the errors per length. We observe a clear distinction between the plots in the figure for both the datasets. This decrease can be attributed to the inclusion of data diversity. Therefore, we observe a significant dip (green plot in Figure 10) in the WA-ECR values over both the Hindi real datasets, which is a clear indication of our models’ superior performance.

**Figure 11:**
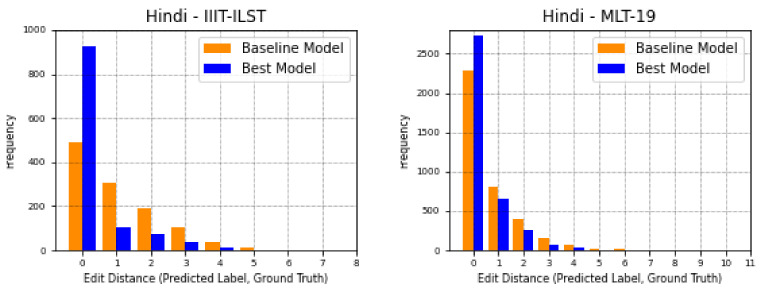

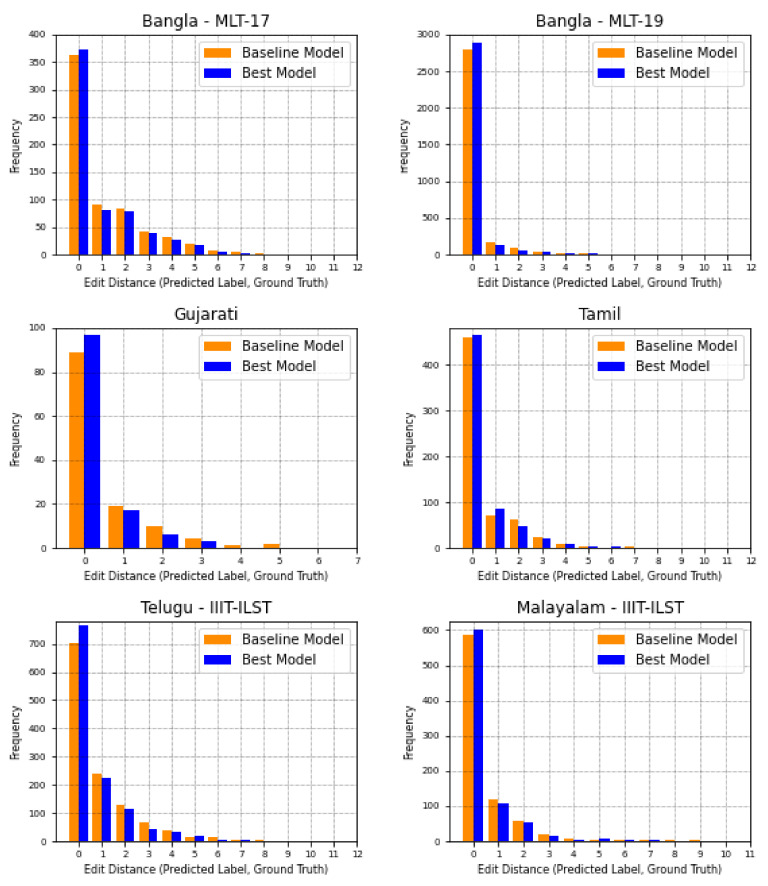
Histogram of correct words (x=0) and words with *x* errors (x>0) for Baseline 2 model as baseline model and the best model trained so far evaluated on real datasets for six languages (Hindi, Bangla, Gujarati (our dataset), Tamil (our dataset), Telugu and Malayalam in row-wise order).

The histogram plots are plotted against the edit distance of the predicted label and ground truth of the test image. Meanwhile, WA-ECR plots are a normalized way of presenting the total number of errors per label length. Both of these are useful in inspecting the overall performance of STR systems. The bars at x=0 position indicated the labels that are predicted correctly, whereas the bars at other positions, x>0 indicate the incorrectly predicted labels with an edit distance of value *x*. As we observe in Figure 11, the performance of the best model for each language over performs the Baseline 2 model at x=0 position in the plots. We observe a significant difference in the histogram bars for Hindi datasets (first row of Figure 11) because of the inclusion of font diversity and augmented samples.

**Figure 12:**
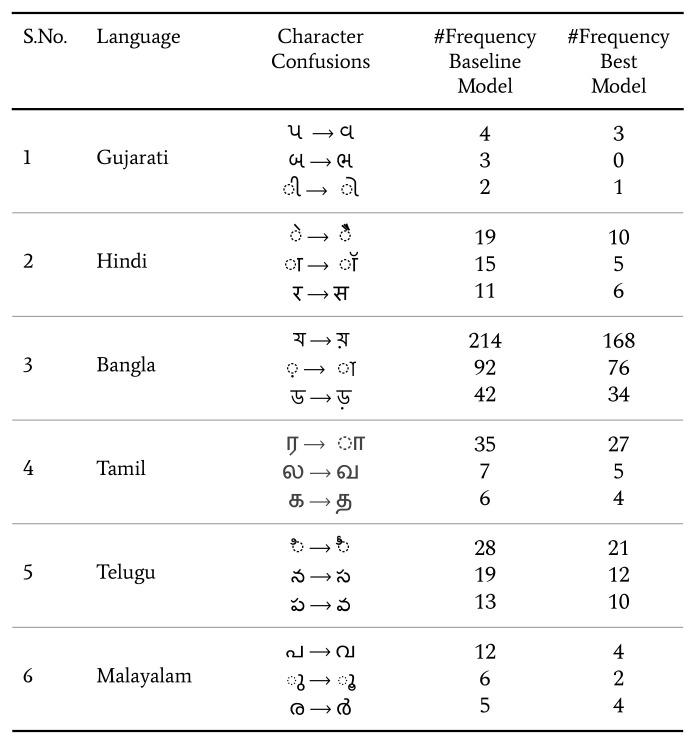
Examples of character confusions over real datasets with models trained on Baseline 2 and the best resultant model trained so far.

We now substantiate how our proposed models, with transfer learning and data diversity improve the detection for top character confusions. We present the example of such confusions in Figure 12. We observe that our best model trained so far is reducing such confusions to a large extent. This also highlights our models’ improved performance over real test sets as compared to the baseline models.

## 8. Conclusions

We created over 2 million synthetic images in six different languages with varying scripts and vocabulary characteristics to improve Scene Text Recognition (STR) for low-resource Indian languages. We analyzed the language transfers for two different baselines via several controlled experiments. The transfer of image and text features seems intuitively relevant for text recognition models in deep learning. However, in this study, we observe that transferring English STR models to Indian languages turned out to be inefficient and, in most cases, resulted in lower-quality models than those trained on individual languages. Our experiments show that transfer learning across the six languages that share a resemblance in terms of glyphs structure and n-gram distributions results in a performance boost compared to the individual baselines. Our STR models performed better than the previous works in all Indian languages, and we have established new standards for recognizing scene-text in low-resource Indian languages. When plugged into the STAR-Net model, the proposed end-to-end trainable Correction BiLSTM improves the Bangla results further while finetuning. For the IIIT-ILST Hindi dataset, we have achieved WRRs similar to English STR by incorporating font diversity and augmentation into our recognition network. We also accomplish the CRRs of above 90% for the two Hindi datasets (IIIT-ILST and MLT-19). Our work in Hindi lays the foundation for boosting the accuracy of non-Latin scene-text recognition systems. Rendering non-Unicode fonts with synthetic generators in other languages remains challenging due to the diligent efforts required to create (non-Unicode to Unicode) text conversion tables and effectively handle the (‘matras’ or vowels in) conjunct characters. For future work in this area, we plan to explore and integrate more fonts in other non-Latin languages, since incorporation of more fonts is one way to enhance data diversity to boost the accuracy of scene-text recognition models. We can curate such variety of fonts by designing font generators using training Generative Adversarial Networks (GANs), which can be modified according to low-resource non-Latin languages like Indian languages.

## Figures and Tables

**Table 1:** Statistics of synthetic data. #, μ, σ represent number of samples, mean and standard deviation.

Language	# Images	Train	Test	μ, σ Word Length	# Fonts
English	17.5 M	17 M	0.5 M	5.12, 2.99	>1200
Gujarati	2.5 M	2 M	0.5 M	5.95, 1.85	12
Hindi	7.5 M	7 M	0.5 M	8.73, 3.10	97
Bangla	2.5 M	2 M	0.5 M	8.48, 2.98	68
Tamil	2.5 M	2 M	0.5 M	10.92, 3.75	158
Telugu	5 M	5 M	0.5 M	9.75, 3.43	62
Malayalam	7.5 M	7 M	0.5 M	12.29, 4.98	20

**Table 2:** Results of individual baselines on synthetic datasets.

		Baseline 1		Baseline 2	
S.No.	Language	CRR	WRR	CRR	WRR
1	English	77.13	38.21	**86.04**	**57.28**
2	Gujarati	94.43	81.85	**97.80**	**91.40**
3	Hindi	89.83	73.15	**95.78**	**83.93**
4	Bangla	91.54	70.76	**95.52**	**82.79**
5	Tamil	82.86	48.19	**95.40**	**79.90**
6	Telugu	87.31	58.01	**92.54**	**71.97**
7	Malayalam	92.12	70.56	**95.84**	**82.10**

**Table 3:** Results of Transfer Learning (TL) on synthetic datasets. Parenthesis contain results on target languages w/o TL. TL among Indic scripts improves Baseline 2 results.

		Baseline 1		Baseline 2	
S.No.	Language	CRR	WRR	CRR	WRR
1	English → Gujarati	92.71 (**94.43**)	77.06 (**81.85**)	97.50 (**97.80**)	90.90 (**91.40**)
2	English → Hindi	88.11 (**89.83**)	70.12 (**73.15**)	94.50 (**95.78**)	80.90 (**83.93**)
3	Gujarati → Hindi	**91.98** (89.83)	73.12 (**73.15**)	**96.12** (95.78)	**84.32** (83.93)
4	Hindi → Bangla	91.13 (**91.54**)	70.22 (**70.76**)	**95.66** (95.52)	**82.81** (82.79)
5	Bangla → Tamil	81.18 (**82.86**)	44.74 (**48.19**)	**95.95** (95.40)	**81.73** (79.90)
6	Tamil → Telugu	87.20 (**87.31**)	56.24 (**58.01**)	**93.25** (92.54)	**74.04** (71.97)
7	Telugu → Malayalam	90.62 (**92.12**)	65.78 (**70.56**)	94.67 (**95.84**)	77.97 (**82.10**)

**Table 4:** Results on real datasets. FT indicates fine-tuned. For number of synthetic training samples and fonts, refer Table 2. # represent the number of samples.

S.No.	Language	Dataset	# Images	Model	CRR	WRR
1				Baseline 1	84.93	72.08
2	Gujarati	ours	125	Baseline 2	88.55	74.60
3				Baseline 2 Eng→Guj	78.48	60.18
4				Baseline 2 Hin→Guj	**91.13**	**77.61**
5				Mathew et al. [4]	75.60	42.90
6				Baseline 1	78.84	46.56
7				Baseline 2	78.72	46.60
8	Hindi	IIIT-ILST	1150	Baseline 2 Eng→Hin	77.43	44.81
9				Baseline 2 Guj→Hin	79.12	47.79
10				OCR-on-the-go [15]	-	51.09
11				Baseline 2 Guj→Hin FT ${}^{1}$	**83.64**	**56.77**
12				Baseline 1	86.56	64.97
13	Hindi	MLT-19	3766	Baseline 2	86.53	65.79
14				Baseline 2 Guj→Hin	**89.42**	**72.66**
15				Bušta et al. [2]	68.60	34.20
16				Baseline 1	71.16	52.74
17	Bangla	MLT-17	673	Baseline 2	71.56	55.48
18				Baseline 2 Hin→Ban	72.16	57.01
19				W/t Correction BiLSTM	**83.30**	**58.07**
20				Baseline 1	81.93	74.26
21	Bangla	MLT-19	3691	Baseline 2	82.80	77.48
22				Baseline 2 Hin→Ban	**82.91**	**78.02**
23				Baseline 1	**90.17**	70.44
24	Tamil	ours	634	Baseline 2	89.69	71.54
25				Baseline 2 Ban→Tam	89.97	**72.95**
26				Mathew et al. [4]	**86.20**	57.20
27	Telugu	IIIT-ILST	1211	Baseline 1	81.91	58.13
28				Baseline 2	82.21	59.12
29				Baseline 2 Tam→Tel	82.39	**62.13**
30				Mathew et al. [4]	**92.80**	73.40
31	Malayalam	IIIT-ILST	807	Baseline 1	84.12	70.36
32				Baseline 2	91.50	72.73
33				Baseline 2 Tel→Mal	92.70	**75.21**

^1^ Fine-tuned on MLT-19 dataset as discussed earlier. We fine-tune all the layers.

**Table 5:** Results of Baseline 2 on Hindi datasets with increase in fonts and augmentations. # represents the number of samples.

S.No.	Dataset	# Synth Images	# Fonts	CRR	WRR
1	IIIT-ILST	7 M	97	78.72	46.60
2		7 M	552	88.00	71.45
3		8 M (Aug)	552	**90.92**	**80.33**
4	MLT-19	7 M	97	86.53	65.79
5		7 M	552	87.88	67.12
6		8 M (Aug)	552	**90.15**	**72.77**

**Table 6:** Results of lexicon-based transcription on real datasets. In the parenthesis, ‘50’ and ‘1 K’ indicate the lexicon sizes.

			Lexicon-Free		Small (50)		Large (1k)	
S.No.	Language	Dataset	CRR	WRR	CRR	WRR	CRR	WRR
1	Gujarati	ours	91.13	77.61	**100.0**	**100.0**	92.20	78.40
2	Hindi	IIIT-ILST	90.92	80.33	99.27	**98.86**	**99.35**	**98.86**
3	Hindi	MLT-19	90.15	72.77	**99.16**	**98.69**	97.37	90.58
4	Bangla	MLT-17	83.30	58.07	**93.38**	**89.95**	87.86	65.62
5	Bangla	MLT-19	82.91	78.02	**97.21**	**95.69**	96.23	89.73
6	Tamil	ours	89.97	72.95	**98.03**	**97.00**	94.40	81.70
7	Telugu	IIIT-ILST	86.20	62.13	**96.49**	**94.63**	89.11	63.33
8	Malayalam	IIIT-ILST	92.70	75.21	**98.41**	**97.52**	93.67	76.08

## Data Availability

Data available in a publicly accessible repository that does not issue DOIs. Data is openly available at Gujarati and Tamil datasets, S.G., R.S., C.V.J., accessed on September 2021.
